# Early growth, development and allometry of glyphosate-resistant and susceptible *Amaranthus palmeri* in response to current and elevated temperature and CO_2_

**DOI:** 10.1038/s41598-023-41121-5

**Published:** 2023-09-02

**Authors:** Juliana de Souza Rodrigues, Donn Shilling, Viktor Tishchenko, Samantha Bowen, Shiyuan Deng, Daniel B. Hall, Timothy L. Grey

**Affiliations:** 1 Department of Crop and Soil Sciences, University of Georgia, 2360 Rainwater Road, Tifton, GA 31793 USA; 2 Department of Crop and Soil Sciences, University of Georgia, 120 Carlton Street, Athens, GA 30602 USA; 3 Department of Crop and Soil Sciences, University of Georgia, 1109 Experiment Street, Griffin, GA 30223 USA; 4 Department of Statistics, University of Georgia, 310 Herty Drive, Athens, GA 30602 USA

**Keywords:** Invasive species, Climate-change impacts, Plant evolution

## Abstract

This study aimed to evaluate the influence of CO_2_ and temperature on glyphosate-resistant and susceptible biotypes of *Amaranthus palmeri* (Palmer amaranth) in terms of morphological development. Height (cm), stem diameter (cm), leaf area (cm^2^), number of leaves, leaf, stem, and root dry matter, plant volume (m^3^), as well as shoot-to-root allometry were evaluated. The Palmer amaranth biotypes were grown under four different scenarios: 1—low temperature (23/33 °C) and CO_2_ (410 ± 25 ppm); 2—low temperature (23/33 °C) and high CO_2_ (750 ± 25 ppm); 3—high temperature (26/36 °C) and low CO_2_ (410 ± 25 ppm); and 4—high temperature (26/36 °C) and CO_2_ (750 ± 25 ppm). Between CO_2_ and temperature, the majority of differences observed were driven by CO_2_ levels. Palmer amaranth grown under 750 ppm of CO_2_ was 15.5% taller, displayed 10% more leaf area (cm^2^), 18% more stem dry matter, and had a 28.4% increase in volume (m^3^) compared to 410 ppm of CO_2_. GA2017 and GA2020 were 18% and 15.5% shorter, respectively. The number of leaves was 27% greater for GA2005. Plant volume decreased in GA2017 (35.6%) and GA2020 (23.8%). The shoot-to-root ratio was isomeric, except at 14 and 21 DAT, where an allometric growth towards shoot development was significant. Palmer amaranth biotypes responded differently to elevated CO_2_, and the impacts of temperature need further investigation on weed physiology. Thus, environmental and genetic background may affect the response of glyphosate-resistant and susceptible populations to climate change scenarios.

## Introduction

Climate change is one of the most significant environmental challenges facing the world today, and it is already having a profound impact on ecosystems and agriculture around the globe. Elevated temperature, rising carbon dioxide (CO_2_)_,_ salinity, and drought affect plant growth and are a threat to agriculture, and can lead to changes in the plant community structure and composition^1^. According to IPCC^2^, the rise in global average temperature tends to obscure the notable temperature differences between land and sea, and between high and low latitudes. In high latitudes, there is a high likelihood of precipitation increases, while in most of the tropic and subtropical land regions, precipitation decreases are expected.

Carbon dioxide (CO_2_) is known as a primary contributor to the greenhouse effect and subsequent temperature increase, but it is also a vital component for plant photosynthesis. When atmospheric CO_2_ levels rise, photosynthesis rates increase in C3 plants, leading to a phenomenon known as CO_2_ fertilization^3^. CO_2_ fertilization could counterbalance some of the effects of temperature increase, particularly in regions where plant growth is constrained by water availability^4^. This may alter the competitive balance between species that differ in their photosynthetic pathways, rooting depths, and other effects^4^. Because of environmental changes, plant communities can be affected by shifts in the geographic range of plant species. In some regions, warmer temperatures have allowed plants to move to higher elevations or latitudes where they previously could not survive^5^. An increase of 1 °C in temperature can move ecological zones by 160 km, and in the northern hemisphere, increases up to 4 °C will move species up to 500 km^6^. At the same time, other plant species could be reduced in abundance or disappear altogether due to changes in their habitat conditions^7^. Changes in CO_2_ concentration and temperature may alter the competitive balance between weeds and crops between different and same photosynthetic pathways (C3/C4), rooting depths^4^, nutrient availability, and extreme weather conditions^8^. According to a recent published review^9^, it is proposed that the impact of weeds on crops under climate change will be comparable in magnitude to their current effects under existing climatic conditions. However, the same authors also highlighted a lack of studies that assess the interactive and individual effects of climate change and weeds on crop varieties within the same experimental conditions. Overall, the capacity of various species to cope with climate change will rely on their ability to follow the changing climate by migrating to new areas or adjusting their physiology to acclimate to the new surroundings^10^.

Increasing temperatures and CO_2_ may also influence the allometric growth of species. Allometry is the quantitative relationship between organs within an individual that grow at different rates^11^. This concept may be used to study organs' size, shape, function and to estimate different metabolic parameters, making allometric relationships valid for different parts of the plant and throughout its life cycle^12,13^. For example, Canada thistle (*Cirsium arvense*) exhibited an increase in the root-to-shoot ratio, with a notable rise in root dry matter under elevated CO_2_ conditions (∼ 350 μmol mol^−1^ above ambient)^14^. Another study, evaluating Sydney golden wattle (*Acacia longifolia* ssp. *Longifoli*a) growth under well-watered conditions and 700 ppm of CO_2_, concluded that root dry weight was enhanced by elevated CO_2_^15^. Allometric growth is based on the allocation of specie biomass and is primarily governed by genotype expression and interaction with environment^11^. Additionally, allometric growth can be influenced by phenotypic traits. Thus, allometric relationships can be used to forecast plant growth, health and ecosystem processes, and serve as a measure of plant plasticity in response to changing environmental conditions^16,17^.

Herbicide-resistant weeds poses a significant threat as they spread across agricultural production regions, and climate change may expand the territories infested by the most troublesome weeds. Palmer amaranth's (*Amaranthus palmeri*) ability to adjust to environmental variations is one reason for its successful introduction and rapid distribution. This weed exhibits a remarkable level of plasticity to various environmental factors, including light, temperature, water availability, and human management practices^18^. Palmer amaranth is a dioecious C4 summer annual native to the Sonoran Desert regions of northern Mexico and the southwestern United States^19^. It began spreading beyond its original habitat in the early 1900s because of human-mediated seed dispersal and the creation of new habitats through agricultural expansion^18,20^.

Palmer amaranth seeds are small and exhibits wind pollination leading to rapid development of herbicide resistance enhancing its survival across various agroecosystems^20^. This specie also has a prolonged germination period that extends throughout the growing season^19,20^, with optimal germination and dry matter production occurring at day and night temperatures of 35/30 °C^21,22^. The emergence of Palmer amaranth can be influenced by management practices such as tillage and herbicides, and may potentially lead to population shifts reported in the literature for species such as *Bassia scoparia* ^23^; horseweed (*Conyza canadensis*)^24^ and common waterhemp (*Amaranthus tuberculatus*)^25^. A single female Palmer amaranth plant can produce up to 600,000 seeds growing isolated, and more than 100,000 seeds in competition with crops^20,26^. Under drought stress, Palmer amaranth survived and produced at least 14,000 seeds plant^−1^^27^. Palmer amaranth seeds grown under limited water conditions demonstrated increased weight, reduced dormancy, and high germination rates^28^. In addition, the sex dimorphism and flowering pattern of Palmer amaranth can be influenced by a range of growing conditions and management practices^28–31^.

The selection pressure imposed by the recurrent use of herbicides with the same mode of action (MoA) resulted in the evolution of herbicide-resistant weeds. Palmer amaranth is one species that has evolved resistance to multiple modes of action^18^. According to the HRAC^32^ mode of action classification, these include inhibitors of the of acetolactate synthase (ALS-2), microtubule assembly (3), auxin mimics (4), PSII inhibitors (5), enolpyruvyl shikimate phosphate synthase (EPSP-9), glutamine synthetase (10), protoporphyrinogen oxidase (PPO-14), very long-chain fatty acid synthesis (15), and hydroxyphenyl pyruvate dioxygenase (27) were related to herbicide-resistance in Palmer amaranth populations worldwide^33^.

Models of herbicide resistance evolution often assume that there is a fitness cost associated with resistance as a result of the genetic variability found within populations. In triazine-resistant weeds with the Ser-264-Gly gene mutation in the catalytic site of D1 protein, besides the resistance to triazine herbicides, the mutation leads to a reduction in the electron transfer rate in the photosystem II, decreasing photosynthesis rates^34^. However, fitness costs are not always observed in herbicide resistant weed populations, and no resistance costs has been detected in glyphosate-resistant Palmer amaranth^35,36^. Furthermore, in cases where fitness costs are present, they have been demonstrated to be influenced by the genetic background of the population^37^. The fitness cost of an adaptive allele can manifest as a direct cost due to the pleiotropic effect of the resistance allele, and via ecological trade-offs in traits such as plant growth, development, and resource partitioning^38^, height, and flowering time^39^. These trade-offs can ultimately lead to a direct fitness cost in an environment with limited resources^36^.

The rise of atmospheric CO_2_ levels and higher temperatures caused by global climate change is expected to expand Palmer amaranth range, increasing the challenge to manage these populations and its adverse impacts on the agriculture practices^40^. Here, we aim to report the isolated and combined effects of elevated CO_2_ and temperature on biotypes of glyphosate-resistant and susceptible Palmer amaranth on plant growth and development. We hypothesized that the Palmer amaranth biotypes will respond differently to CO_2_ and temperatures variations, and allometric shoot to root relationship will also be affected.

## Materials and methods

### Plant material

Seeds of three different populations of Palmer amaranth GA2005, GA2017, and GA2020 were collected at Tift, Bibb and Sumter counties, respectively in Georgia, US. Seeds were then stored under dry and cold conditions until use^41^. Seeds were sown in trays filled with potting media (Pro-Mix^®^, BX, Quebec, Canada). The greenhouse was maintained at 30 °C ± 5 °C, and natural light was supplemented for 12 h each day by metal halide lamps (400 µE m^−2^ s^−1^), and relative humidity ranging from 40 to 70%.

### Dose–response assessment

The Palmer amaranth population were submitted to a dose–response screening to determine whether or not the populations are susceptible or resistant to glyphosate, flumioxazin, atrazine, and imazapic. These herbicides were chosen based on their use in crops grown in Georgia, US. Seeds were planted separately in square pots (9 × 9 cm) filled with Tifton loamy sand^42^. Seedlings were thinned to one plant per pot within 4 d after emergence. Plants received irrigation twice a day and fertilization as needed to maintain growth. The experiment was repeated three times in a complete randomized design with three replicates per treatment, and the methodology was adapted^43^ to include doses ranging from 1/16× to 16× times the recommended dose for all herbicides tested (data not shown).

### Growth chambers experiment

After achieving the desired height (8 to 10 cm) growing at the greenhouse, seedlings were transplanted into 5-L round containers filled with potting media (Pro-Mix^®^, BX, Quebec, Canada). Fertilizer Osmocote Blend, 18-5-12 (ICL^®^ Specialty Fertilizers, Holland) was added, and the containers were placed inside walk-in growth chambers (model CG72, Conviron^®^, Winnipeg, Canada) located at the Georgia Envirotron, Griffin Campus, GA. The growth chambers were scheduled to operate under four scenarios: 1—23/33 °C, 410 ± 25 ppm; 2—23/33 °C, 750 ± 25 ppm; 3—26/36 °C, 410 ± 25 ppm and 4—26/36 °C, 750 ± 25 ppm. These scenarios represent low/high temperatures (23/33 °C and 26/36 °C; night/day) and low and high CO_2_ concentrations (410 and 750 ppm) combined. The increases in temperatures and CO_2_ levels evaluated in this study were derived from projected future scenarios outlined by the Intergovernmental Panel on Climate Change^2^. The base temperature employed was determined by averaging the highest and lowest temperatures observed during the summer season in Central and South Georgia, US, where the biotype seeds were collected. The lighting in the growth chambers was adjusted to provide a light intensity of 700 µmol/m^2^/s, following a 16-h day and 8-h night photoperiod. The plants were fertilized on a weekly basis, and drip irrigation was scheduled for 15 min, twice a day.

### Study design

The study consisted of a full factorial structure with four factors, in a randomized complete block design, with three replicates. The treatment factors considered were temperature (23/33 °C and 26/36 °C), CO_2_ levels (410 and 750 ppm), biotypes (GA2005, GA2017 and GA2020) and harvest dates (14, 21 and 28 days after transplant, DAT) (Table 1). For analysis, the scenarios involving combinations of CO_2_ and temperature in the growth chambers were treated separately. This separation was done to specifically to assess and identify the individual impacts of CO_2_ and temperature on biotype and DAT. The experiment was conducted in 2021 and 2022, with a total of 216 plants and year was considered a blocking factor.

**Table 1:** Combination of treatment factors temperature, CO_2_, biotype, days after transplant (DAT) and year tested for Palmer amaranth, and their respective levels.

Factors	Levels
Temperature	23/33 °C and 26/36 °C (night/day)
CO_2_	410 and 750 ppm, ± 25 ppm
Biotype	GA2005, GA2017 and GA2020
DAT	14, 21 and 28
Year	2021 and 2022

Year was considered a blocking factor on the statistical analysis.

### Data collection (growth parameters)

At each harvest date, the height (cm), widest horizontal diameters 1 and 2 (cm), stem diameter (mm), number of leaves, leaf area (cm^2^), leaf dry matter (g), stem matter (g), root dry matter (g) and plant volume (cm^3^) were recorded. The plant volume was calculated the following formula^44^:

$$elliptical \; column = height*\frac{1}{2}diameter \, 1*\frac{1}{2}diameter \, 2*\pi $$

For each plant, leaves, stems, and roots were separated. The number of leaves was counted, and the foliar area (cm^2^) measured using the LI-3100C area meter (LI-COR^®^, Lincoln, NE). Roots were hand washed carefully, and the plant parts were placed in separated paper bags. The samples were placed in oven with forced air circulation (60 °C) until constant dry matter was achieved and then weighed to determine the final dry matter (g). Above-ground dry matter (stems and leaves) and root dry matter were used to analyze plant allometry under the scenarios tested.

### Data analysis

#### Dose–response

The data obtained from the dose–response experiments were submitted to an analysis of variance evaluating population (GA2005, GA2017 and GA2020) and herbicide dose ($\frac{1}{16}D, \frac{1}{8}D,\frac{1}{2}D, \frac{1}{4}D$, D, 2D, 4D, 8D, 16D), with D (dose) being the recommended dose of each herbicide in g/ha^−1^. Each herbicide was evaluated separately and model selection was based on the lack-of-fit F test^45^. The raw data points from each population were subsequently fitted to a four-parameter log-logistic function^45^:

$$y=c+ \frac{d-c}{1+\mathrm{exp}(b\left(\mathrm{log}\left(x\right)-\mathrm{log}\left(e\right)\right))}$$

where *y* is shoot dry weight as a percentage of untreated control, x is herbicide dose in g/ha^−1^, *c* is the lower response limit, *d* is the upper limit, *b* is the slope, and *e* is the ED_50_, the herbicide dose that causes 50% reduction in shoot dry weight. Data was analyzed using *drc* package^46,47^ in RStudio^48^.

#### Plant growth and development

Table 1 displays the experimental factors and their levels. Preliminary univariate analyses were carried out to identify any non-normality and non-constant variance in each response variable. Corrective log or square-root transformations were applied as necessary. The resulting eight response variables were then analyzed jointly by fitting a multivariate analysis of variance model appropriate for the study design^49^. This model incorporated main effects and all interactions among the experimental factors, with the main effects of year, the blocking factor, and all interactions involving year treated as random. The model was simultaneously fitted to all eight responses, assuming normal errors that were independent across distinct experimental units but with an unstructured 8 by 8 covariance matrix for the vector-valued response on each unit.

The interactions and main effects from the multivariate model were of primary interest, but secondary univariate analyses were also conducted to detect significant main effects and interactions for individual response variables. Due to the repeated testing of these effects on eight responses, a Bonferroni correction was applied to all tests conducted in the univariate analyses. The significance levels for these tests were all divided by eight. Significant interactions were evaluated via interaction plots. For factors that were not involved in significant interactions but had significant main effects, pairwise contrasts were tested. In the case of DAT, all pairwise contrasts were tested with Tukey HSD-corrected p-values, and for biotype, pairwise contrasts with the susceptible biotype were tested with a Dunnett correction. No multiplicity correction was necessary for two-level factors CO_2_ and temperature. All multiplicity-adjusted p-values were compared to the Bonferroni-corrected significance level of 0.0062 to determine statistical significance. The analyses were done in R using the nlme^50^, lme4^51^, lmerTest^52^, and car^53^ packages in RStudio^48^. Large sample Wald tests are reported for the multivariate analysis. Kenward-Roger adjusted approximate F tests are reported for univariate analyses.

Next, the allometric relationships between shoots (leaves plus stem) (y) and roots (x) dry matter (g) were tested to for all biotypes and every treatment factor. Linear regression aims to minimize the distance between the observed values and the regression line in the y-direction. As such, it is well-suited for predicting the value of one variable based on another variable. However, since measurement errors can occur in x and y, minimizing the sum of squared deviations in the y-direction is not ideal. In contrast, the standardized major axis (SMA) estimation method determines the minimum distance between the observed values and the regression line while considering the deviations in both x and y directions, as well as the slope of the variables. This makes SMA more appropriate for estimating the slope of the allometric scaling equation. The SMA regression was utilized to establish the correlation between log-transformed shoot and root dry matter. The allometric relationship was represented by the equation log y = log b + a * log x, where 'a' denotes the scaling exponent (slope) and 'b' represents the allometric coefficient or "scaling factor" (y-intercept/elevation). The standardized major axis regression (SMA), also known as reduced major axis (RMA), was utilized to evaluate differences in shifts of the slope and elevation of slopes (y-intercept). The ‘smatr’ package^54^ in RStudio^48^ was used to obtain the SMA slopes, intercepts, and its confidence interval (95%). The allometric analysis was used to mainly verify whether or not the biomass portioning (shoot to root ratio) changes among biotypes under the treatment factors tested.

### Permissions required

The authors collected GA2005 and GA2020 seeds used, and Dr. Stanley Culpepper, UGA Tifton, collected GA2017. All seeds were gathered from experimental fields associated with Research and Extension with the University of Georgia.

### Guidelines required

The collection of Palmer amaranth seeds used in this study complies with the University of Georgia institutional guidelines.

## Results

For the dose–response assessment, the populations tested were resistant only to glyphosate. We determined the ED_50_, which is the dosage that reduces 50% in shoot dry weight. Biotype GA2005 was considered susceptible (ED_50_ = 272 a.e. g/ha) to glyphosate, and biotypes GA2017 and GA2020 were considered glyphosate-resistant (ED_50_ = 1180 a.e. g/ha, and ED_50_ = 3603 a.e. g/ha, respectively). Results were based on the recommended dose of 832 g a.e. g/ha of glyphosate^43^.

According to the multivariate model, the analysis of growth and development indicated that CO_2_ levels, biotype, and DAT (p < 0.001) were significant, whereas temperature did not show significant effects. No interaction was observed (Table 2).

**Table 2:** Results from the fitted multivariate model.

Treatment factors	Df	Chisq	Pr (> Chisq)
Temperature	8	9.22	0.3238
CO_2_	8	35.2	** < 0.001**
Biotype	16	46.64	** < 0.001**
Days after transplant (DAT)	16	1171.27	** < 0.001**
Temperature * CO_2_	8	12.37	0.1351
Temperature * biotype	16	12.83	0.6851
Temperature * DAT	16	19.43	0.2469
CO_2_ * biotype	16	12.90	0.6801
CO_2_ *DAT	16	17.04	0.3830
Biotype * DAT	32	21.32	0.9243
Temperature * CO_2_ * biotype	16	9.72	0.8805
Temperature * CO_2_ * DAT	16	21.35	0.1653
Temperature * biotype * DAT	32	22.61	0.8903
CO_2_ * biotype * DAT	32	22.72	0.8869
Temperature * CO_2_ * biotype * DAT	32	12.53	0.9992

The analysis showed the CO_2_, biotypes, and days after transplant (DAT) as main effects on the growth and development of Palmer amaranth.

Significant values are in bold.

After evaluating the global tests from the multivariate model, univariate analyses were conducted to understand how the treatment factors affect the response variables of height (cm), diameter (cm), number of leaves, leaf area (cm^2^), leaf, stem, roots dry matter (g) and plant volume (m^3^) (Table 3). Data related to DAT can be found in the supplementary information section.

**Table 3:** P-values of the ANOVA test for the eight response variables tested.

Treatment factors	Height (cm)	Diameter (cm)	Number of leaves	Leaf area (cm^2^)	Leaf dry matter (g)	Stem dry matter (g)	Roots dry matter (g)	Plant volume (m^3^)
	p-value							
Temperature	0.4136	0.6994	0.1078	0.0338*	0.7675	0.3881	0.8635	0.1450
CO_2_	** < 0.0062**	0.0104*	0.0090**	** < 0.0062**	0.1254	** < 0.0062**	0.1711	** < 0.0062**
Biotype	** < 0.0062**	0.7602	** < 0.0062**	0.8200	0.4767	0.0159*	0.9686	** < 0.0062**
Days after transplant (DAT)	** < 0.0062**	** < 0.0062**	** < 0.0062**	** < 0.0062**	** < 0.0062**	** < 0.0062**	** < 0.0062**	** < 0.0062**
Temperature * CO_2_	0.1252	0.0189*	0.8121	0.5741	0.5798	0.0557	0.0623	0.1245
Temperature * biotype	0.6948	0.7638	0.3445	0.0823	0.6772	0.8058	0.6146	0.7088
Temperature * DAT	0.8738	0.9527	0.3444	0.4308	0.6063	0.9315	0.4344	0.9571
CO_2_ * biotype	0.5471	0.6345	0.0169*	0.4281	0.3803	0.6518	0.5251	0.3425
CO_2_ *DAT	0.8948	0.5812	0.9140	0.5639	0.1356	0.4466	0.1936	0.3770
Biotype * DAT	0.4466	0.9083	0.3966	0.5232	0.6404	0.9215	0.1955	0.4013
Temperature * CO_2_ * biotype	0.6466	0.6179	0.3778	0.6380	0.2822	0.9402	0.9863	0.5520
Temperature * CO_2_ * DAT	0.3368	0.6638	0.0499*	0.5465	0.4779	0.9313	0.1452	0.5299
Temperature * biotype * DAT	0.6780	0.6115	0.6473	0.9649	0.5231	0.4712	0.8875	0.4610
CO_2_ * biotype * DAT	0.9282	0.8378	0.9544	0.2930	0.5476	0.7406	0.0869	0.7350
Temperature * CO_2_ * biotype * DAT	0.9290	0.9684	0.9122	0.8625	0.5571	0.9434	0.5880	0.9719

The Bonferroni adjusted level of significance considered is < 0.0062. The interactions observed for diameter (cm) (Temperature * CO_2_), number of leaves (CO_2_ * biotype and Temperature * CO_2_ * DAT), roots dry matter (CO_2_ * biotype * DAT), as well the main effects with p-value less than 0.05 were not considered statistically significant based on a Bonferroni-adjusted threshold of 0.05/8 = 0.0062. Signif. codes: ‘***’ 0.001 ‘**’ 0.01 ‘*’ 0.05.

Significant values are in bold.

### CO_2_ levels

The study revealed noteworthy primary effects of CO_2_ on plant characteristics including mean plant height (cm), leaf area (cm^2^), stem dry matter (g) and plant volume (m^3^). Specifically, plants cultivated under 750 ppm displayed a 15.5% increase in height, a 10% increase in leaf area, an 18% increase in stem dry matter, and a 28.4% increase in volume compared to those grown under 410 ppm (Table 4).

**Table 4:** Marginal means and significant effects of CO_2_ in height (cm), leaf area (cm^2^), stem dry matter (g) and plant volume (m^3^) in Palmer amaranth.

Response variables	CO_2_ level (ppm)	Response	SE	p-value
Height (cm)	410	60.0	± 3.12	0.0011
	750	69.3	± 3.60	
Leaf area (cm^2^)	410	2216	± 532	0.0057
	750	2432	± 584	
Stem dry matter (g)	410	13.9	± 2.93	0.0014
	750	16.4	± 3.45	
Plant volume (m^3^)	410	0.1346	± 0.0092	* < 0.0001*
	750	0.1879	± 0.0130	

Bonferroni-adjusted intervals statistically significant at a p-value of 0.0062 were used. SE: standard error.

It is worth examining the effect of CO_2_ on Palmer amaranth at the early stages of growth. Plants were transplanted at the 3 to 4 leaf stage and moved to the growth chambers. At the first harvest date (14 DAT), mean height (cm) and leaf area (cm^2^) were 46.3 cm and 1947 cm^2^, respectively.

### Biotypes

GA2005 was taller (72.4 cm) than GA2017 (59.2 cm) and GA2020 (62.6 cm). This represents a decrease of 18% and 15.5% in height for both resistant biotypes. The number of leaves for GA2005 was 217, representing an increase of 27% in comparison to both glyphosate-resistant biotypes. Additionally, the plant volume, which measures the plant's overall architecture, decreased by 35.6% (0.1298 m^3^) and 23.8% (0.1537 m^3^) for GA2017 and GA2020, respectively. The average volume for GA2005 was 0.2017 m^3^ (Table 5).

**Table 5:** Marginal means for biotypes (GA2005, GA2017 and GA2020) in height (cm), number of leaves, and plant volume (m^3^) in Palmer amaranth.

Response variables	Biotypes	Response	SE	Pairwise comparison	p-value
Height (cm)	GA2005	72.4	± 4.09	GA2017 vs. GA2005	*0.0005*
	GA2017	59.2	± 3.39		
	GA2020	62.6	± 3.53	GA2020 vs. GA2005	> 0.00062
Number of leaves	GA2005	217	± 13.9	GA2017 vs. GA2005	*0.0002*
	GA2017	171	± 11.0		
	GA2020	172	± 11.0	GA2020 vs. GA2005	*0.0003*
Plant volume (m^3^)	GA2005	0.2017	± 0.0160	GA2017 vs. GA2005	* < 0.0001*
	GA2017	0.1298	± 0.0103		
	GA2020	0.1537	± 0.0132	GA2020 vs. GA2005	> 0.00062

Bonferroni-adjusted intervals statistically significant at a p-value of 0.0062 were used. The table also shows the results of the Dunnett pairwise contrasts of Palmer amaranth biotypes (GA2005, GA2017, GA2020) for height (cm), number of leaves and plant volume (m^3^). Bonferroni-adjusted intervals statistically significant at a p-value of 0.0062 were used. SE: standard error.

Italic indicate values less then < 0.0062 are significant.

To evaluate the differences in biotype response (Table 5), the Dunnett-adjusted pairwise contrasts with GA2005 were conducted. GA2017 (p < 0.0062) exhibited statistically significant differences in plant height (cm) and volume (m^3^) compared to GA2005. While GA2020 measured shorter and with less volume, it did not meet the significance threshold (*p-value* = 0.0062).

### Allometry

To test the hypothesis of changes between shoot and root ratio among biotypes and across CO_2_, temperature, and DAT, an allometric analysis was performed.

#### *Biotypes and CO*_*2*_

Shoot to root biomass was positively correlated to CO_2_ levels and biotypes tested (P < 0.001) (Table 6). The SMA slopes for CO2 of 410 ppm and 750 ppm were not significantly different to 1 (P > 0.05) for biotypes, indicating isometric growth.

**Table 6:** R^2^, standardized major axis (SMA) slope.

CO_2_ level	Biotype	R^2^	SMA slope + (95% ci)	P (H_0_:slope = 1.0)	Common slope	Elevation + (95% ci)
410 ppm	GA2005	0.70**	0.8659 (0.7214–1.0394)	0.153	0.9044	0.3230 (0.2037–0.4422)
	GA2017	0.86**	0.9028 (0.7892–1.0328)			0.2939 (0.1846–0.4031)
	GA2020	0.76**	0.9358 (0.7854–1.1151)			0.3503 (0.2384–0.4622)
750 ppm	GA2005	0.64**	1.0749 (0.8882–1.3007)	0.303	0.9601	0.3168 (0.2066–0.4270)
	GA2017	0.80**	0.8986 (0.7877–1.0251)			0.2575 (0.1536–0.3615)
	GA2020	0.82**	0.9621 (0.8613–1.0746)			0.2725 (0.1726–0.3724)

P (p-value regarding the biomass allocation relationship, being significant indicating allometric growth ≠ 1 or not significant, isometric growth = 1), common slope and elevation (interception) of biotypes compared between 410 and 750 ppm of CO_2_ and the Palmer amaranth biotypes tested. ** indicate significant differences among biotypes with P < 0.001.

#### Biotypes and DAT

Shoot to root biomass was positively correlated with DAT (Table 7) for all biotypes tested (R^2^, P < 0.001). The SMA slopes for 14 DAT and 21 DAT were significantly different to 1 (P < 0.05) for biotypes, indicating allometric growth. Whereas at 28 DAT, all biotypes showed isometric growth P > 0.05. During 14 DAT and 21 DAT, plants invested more biomass on shoot development, with common slopes of 0.5186 and 0.7590, respectively.

**Table 7:** R^2^, standardized major axis (SMA) slope.

DAT	Biotype	R^2^	SMA slope + (95% ci)	P (H_0_:slope = 1.0)	Common slope	Elevation + (95% ci)
14	GA2005	0.52**	0.5516 (0.3957–0.7689)	0.0000	0.5196	0.6775 (0.5714–0.7837)
	GA2017	0.39**	0.4351 (0.2849–0.6647)			0.6492 (0.5455–0.7529)
	GA2020	0.54**	0.5522(0.3984–0.7653)			0.6542 (0.5470–0.7614)
21	GA2005	0.35**	0.9703 (0.7021–1.3408)	0.0026	0.7590	0.5019 (0.3279–0.6758)
	GA2017	0.68**	0.8399 (0.6316–1.1169)			0.4694 (0.3059–0.6329)
	GA2020	0.75**	0.6307(0.4996–0.7963)			0.5124 (0.3568–0.6680)
28	GA2005	0.70**	1.1175 (0.7211–1.7319)	0.54374	1.1271	0.09272 (-0.2817 to 0.4672)
	GA2017	0.51**	1.2772 (0.8969–1.8188)			0.0032 (-0.3758 to 0.3824)
	GA2020	0.36**	0.9971 (0.7138–1.3929)			0.0706 (-0.3031 to 0.4444)

P (p-value regarding the dry matter allocation relationship, being significant indicating allometric growth ≠ 1 or not significant, isometric growth = 1), common slope and elevation (interception) of biotypes compared between days after transplant (DAT) and Palmer amaranth biotypes tested. **Indicate significant differences among biotypes with P < 0.001.

#### Biotypes and temperature

Dry matter of shoot to root ratio was positively correlated for all biotypes at 23/33 °C and 26/36 °C and SMA slopes were not statistically different from 1 (P > 0.05), with an isometric growth under both temperatures (Table 8). Overall, no differences among biotypes were detected and the treatment factors did not affect the allometric/isometric relationship on Palmer amaranth.

**Table 8:** R^2^, standardized major axis (SMA) slope.

Temperature	Biotype	R^2^	SMA slope + (95% ci)	P (H_0_:slope = 1.0)	Common slope	Elevation + (95% ci)
23/33 °C	GA2005	0.63**	1.0609 (0.8715–1.2915)	0.1086	0.9069	0.3627 (0.2486–0.4769)
	GA2017	0.84**	0.8396 (0.7537–0.9353)			0.3204 (0.2240–0.4167)
	GA2020	0.76**	0. 9322 (0.8114–1.0711)			0.3358 (0.2394–0.4322)
26/36 °C	GA2005	0.69**	0.9069 (0.7678–1.0712)	0.3629	0.9811	0.2458 (0.1219–0.3697)
	GA2017	0.83**	1.0330 (0.8863–1.2040)			0.1993 (0.0799–0.3187)
	GA2020	0.80**	0.9842 (0.8473–1.1434)			0.9842 (0.1421–0.3773)

P (p-value regarding the biomass allocation relationship, being significant indicating allometric growth ≠ 1 or not significant, isometric growth = 1), common slope and elevation (interception) of biotypes compared between 23/33 °C and 26/36 °C and Palmer amaranth biotypes tested. **Indicate significant differences among biotypes with P < 0.001.

## Discussion

This study aimed to investigate the early growth and development of both glyphosate resistant and susceptible Palmer amaranth biotypes in varying CO_2_ and temperature conditions. Additionally, we explored the allometric relationships between the biotypes and the treatment factors, CO_2_, temperature, as well as the DAT. These analyses can provide valuable insights into how biotypes adapt to various environmental stresses^9^.

Height (cm), leaf area (cm^2^), stem dry matter and plant volume (m^3^) were the variables mostly impacted by the increase in CO_2_. Plants can detect a change in atmospheric CO_2_ levels mainly through tissues that are exposed to the open air, which are mostly limited to the plant's photosynthetic organs. The protective layer covering these organs, known as the cuticle, restricts direct exposure of the guard cells of stomata and the mesophyll to changes in atmospheric CO_2_^55^. One of the hypotheses that explains why C4 plants responds to increases in CO_2_ levels in a short term is related to their water use efficiency (WUE). For C4 plants, the water loss is costly for the carbon balance in the plant, so species tend to operate at a low transpiration rate (E) preventing hydraulic failure^56^. In addition, under elevated CO_2_, the WUE is explained by a decrease in 20% of stomatal conductance^55^ which may affect leaf thermoregulation during heat stress. There has been a suggestion that elevated CO_2_ could enhance the WUE of C4 and C3 species by reducing their transpiration rate and boosting their CO_2_ assimilation rate^57^. Conversely, in C4 species, the benefits of increased CO_2_ on photosynthesis could be particularly significant during times of drought, being able to produce more dry matter, and root growth compared to C3 species^55^. Even in the absence of drought, WUE improvement was observed for *Amaranthus retroflexus* and *Amaranthus hypochondriacus, C4 plants*^58^.

Studies reported no changes in plant height while aboveground biomass of winter wheat, a C3 plant, increased under 712 μmol mol^−1^ of CO_2_^57,59^. Whereas for maize and sorghum, C4 plants, grown under well-watered conditions, plant height, leaf area, and biomass of leaf, stem and total above-ground were not affected by elevated CO_2_ at 720 μmol mol^−1^^60^. The response to elevated CO_2_ varies substantially more within C4 plants^57^. Other studies showed an increase in biomass of C4 plants, leading to a conclusion that not only the photosynthetic mechanism can explain the response under elevated CO_2_^61^. In terms of leaf area, the increase observed could be related to cell expansion, due to the increased carbohydrate substrate availability^62–65^.

It has been proposed that alterations in plant physiological metabolism might influence the translocation and accumulation of nutrients, ultimately impacting soil nutrient dynamics amidst future climate changes^66,67^. Besides carbon (C), nitrogen (N) availability is expected to play a pivotal role in determining the influence of CO_2_ on dry matter accumulation^68^. It indicates that the availability of nutrients and resources could profoundly affect photosynthesis and plant growth^69^. In this study, plants were provided with optimal water and nutrient supply to create an ideal growing environment and avoid introducing any additional sources of stress. This factor may have influenced the observed results.

In this study, the susceptible GA2005 exhibited notably greater height (cm), plant volume (m^3^), and number of leaves compared to the resistant biotypes (GA2017 and GA2020). Glyphosate-resistant Palmer amaranth populations from Florida and Georgia showed variations in multiple characteristics such as plant height, days to flowering, fresh and dry matter, and leaf and canopy shape when compared to glyphosate susceptible under current levels of CO_2_^70^. Interestingly, certain traits like growth rate, plant height, dry matter, photosynthetic rate, inflorescence length, pollen viability, and seed set may not show any differences, even in a glyphosate-resistant Palmer amaranth population with approximately 100 EPSPS genes^3^. It appears that herbicide resistance mutations may sometimes lead to changes in weed morphology, development, or phenology, without directly impacting the overall plant fitness^71^. These trait alterations could be attributed to subtle pleiotropic effects of resistance mutations or the coevolution of resistance with non-resistance traits^36,72–75^, in response to diverse selective pressures in agroecosystems. Importantly, it should be noted that these changes in life history traits might not always be expressed under certain environmental conditions. Thus, the genetic background of the populations in this study will be evaluated and made available as a follow-up to the present research.

Plant shoot and root, despite being complementary and interdependent, exhibit distinct rates and magnitudes of response to environmental changes^76^. The allometry analysis shows that besides the allometric growth towards shoot development observed at 14 and 21 DAT, the overall isomeric growth recorded demonstrate no differences in terms of carbon portioning among biotypes when compared to CO_2_, and temperature. In some studies, the below growth can be enhanced by CO_2_^75,77^, but neither root biomass nor shoot to root ratios were affected by CO_2_ in this study. Even though biotypes demonstrated differences related to above ground characteristics, shoot to root ratio was not affected by whether glyphosate resistance is involved or not.

The impact of temperature was found to be statistically insignificant on the morphological traits evaluated on this study. However, it's important to note that temperature plays a vital role in photosynthesis, affecting various aspects such as the electron transport system, photosystems, pigments, photosynthesis-related enzyme activities, gas exchange, chlorophyll fluorescence, membrane thermostability, and osmotic regulation in plants^78^. These effects, in turn, have a significant influence on plant growth and development.

Considering that CO_2_ levels and temperature changes, varies along with other climatic factors, vegetation models have been employed to predict species distribution shifts into new areas. CLIMEX modeling and data from the 1981 to 2010 global climatological dataset were used to project the worldwide distribution of Palmer amaranth^40^. The findings suggest a higher risk of Palmer amaranth establishment in Australia and Africa, with potential for expansion into northern Europe and Canada. In the United States, changes in the timing and intensity of rainfall, coupled with rising temperatures indicate that Palmer amaranth may have greater competitive advantage over warm-season crops.

In summary, the study's findings revealed that CO_2_ had the most pronounced influence on plant height, leaf area, stem dry matter, and plant volume, with greater effects observed at 750 ppm compared to 410 ppm. Distinctions were also observed between susceptible and resistant biotypes, with the glyphosate-susceptible (GA2005) exhibiting greater height, plant volume, and number of leaves compared to glyphosate-resistant biotypes (GA2017 and GA2020). Allometric analysis indicated no variations in carbon partitioning among biotypes concerning CO_2_ and temperature. However, significant allometric growth towards shoot development was observed at 14 and 21 DAT. Nevertheless, to comprehensively assess the impact and aid in management strategies, further studies on physiology, genetic background and crop-weed interaction are essential to elucidate the behavior of Palmer under future scenarios.

### Supplementary Information

Supplementary Figure 1.

## Data Availability

The datasets generated during and/or analyzed during the current study are available from the corresponding author on reasonable request.
