# Supplementary material for: Early growth, development and allometry of glyphosate-resistant and susceptible Amaranthus palmeri in response to current and elevated temperature and CO2
Source: Sci Rep. 2023 Sep 2;13:14427. doi: 10.1038/s41598-023-41121-5 (PMC10475059; doi:10.1038/s41598-023-41121-5)
Supplement: Supplementary file 1 — Supplementary Figure 1. [file 41598_2023_41121_MOESM1_ESM.docx]

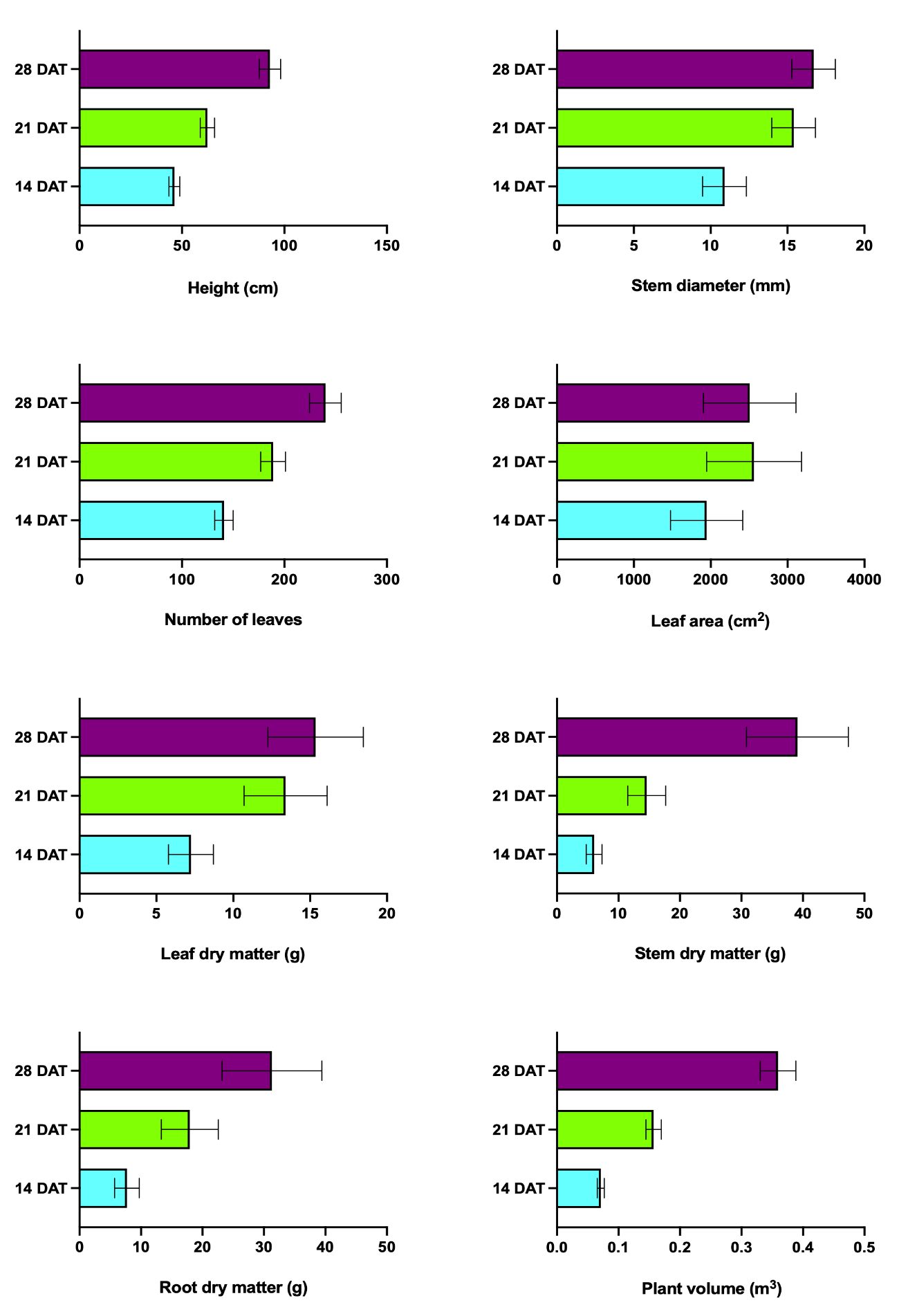


**Figure 1**.Marginal means for Palmer amaranth biotypes (GA2005, GA2017 and GA2020) response at 14, 21 and 28 DAT (days after transplant). Horizontal bars represent the standard error of the mean. Bonferroni-adjusted intervals statistically significant at a p-value of 0.0062 were used.
